# Self-reported symptoms of depression, anxiety and stress in Portuguese primary school-aged children

**DOI:** 10.1186/s12888-020-02498-z

**Published:** 2020-02-27

**Authors:** Diogo Costa, Marina Cunha, Cláudia Ferreira, Augusta Gama, Aristides M. Machado-Rodrigues, Vítor Rosado-Marques, Helena Nogueira, Maria-Raquel G. Silva, Cristina Padez

**Affiliations:** 1 Research Centre for Anthropology and Health, Department of Life Sciences, University of Coimbra, Calçada Martim de Freitas, Edifício São Bento, 3000-456 Coimbra, Portugal; 2 CINEICC - Centre for Research in Neuropsychology and Cognitive and Behavioral Intervention, Faculty of Psychology and Educational Sciences, University of Coimbra, Coimbra, Portugal; 3 Department of Animal Biology, Faculty of Sciences, University of Lisbon, Lisboa, Portugal; 4 High School of Education, Polytechnic Institute of Viseu, Viseu, Portugal; 5 Faculty of Human Kinetics, University of Lisbon, Lisboa, Portugal; 6 Department of Geography and Tourism, University of Coimbra, Coimbra, Portugal; 7 Faculty of Health Sciences, University Fernando Pessoa, Porto, Portugal

**Keywords:** Depression, Anxiety, Stress, Determinants, Children, Primary-school

## Abstract

**Background:**

Symptoms of depression, anxiety and stress experienced during childhood might have a negative impact on development. This study explores factors associated with such symptoms among Portuguese primary school-aged children.

**Methods:**

A sample of children (*n* = 1022, mean age = 8.77 years old) was recruited in public and private schools from the cities of Coimbra, Lisbon and Porto, Portugal. The children’s version of the Depression, Anxiety and Stress Scale (DASS-C) was self-administered. Multivariate logistic regression models were fitted to measure associations (expressed as Odds Ratio, OR and 95% Confidence Intervals, CI) between each DASS-C subscale, dichotomized by its 4th vs. 3rd or less quartiles (symptoms increase with scores), and covariates: child sex, age, socioeconomic status (SES), sports activity beyond school, children self-assessed health status, child and mother’s body mass index and mother’s DASS scores.

**Results:**

Age was negatively associated with anxiety symptoms (adjusted OR, 95% CI: 0.70, 0.57–0.87) and girls, compared to boys, presented lower odds of depressive and stress symptoms (adjusted OR, 95% CI: 0.65, 0.47–0.92 and 0.57, 0.41–0.80, respectively). A low socioeconomic status was associated with more frequent symptoms of stress (adjusted OR, 95%CI for low compared to high SES: 1.61, 1.01–2.56). Children with poorer self-assessed health status and whose mothers scored higher in the DASS also presented significantly higher odds of scoring in the 4th quartile (vs. 3rd or less) of the three DASS-C subscales.

**Conclusions:**

These results suggest the need to tailor preventive efforts targeting childhood mental health symptoms.

## Background

Childhood and adolescent mental health problems represent a growing public health concern that may have long-lasting negative effects [1]. A previous meta-analysis of studies documenting the prevalence of mental disorders in childhood and adolescence, found a worldwide (27 countries) prevalence of 6.5% for any anxiety disorder (95% Confidence Interval: 4.7–9.1), and of 2.6% for any depressive disorder (95% Confidence Interval: 1.7–3.9) [2].

Across Europe, there is a substantial number of children with unmet needs for mental health care [3], although studies documenting the magnitude and factors associated with the most common mental health problems, particularly in younger ages, are limited. There are no recently published prevalence estimates of mental health problems in such young ages in Portugal. There is, however, indication of an increase (by 23%) in the number of children attending outpatient psychiatric consultations, between 2011 and 2013 in the country, and an increase of 30% in the number of new consultations [4].

Depression and anxiety symptoms among young children can have a negative impact on their development [5, 6], affecting their quality of life [7] and constitute a risk factor for poor academic performance and poor social functioning [8].

Even at low thresholds, depressive and anxiety symptoms may represent an increased risk for the development of mental disorders in adulthood, particularly, depression [7, 9]. Several mental health problems observed during adulthood might have their origin in childhood and adolescence adverse experiences [10–12]. Longitudinal evidence also supports that social factors can play a significant role in determining mental health problems during childhood and adolescence [13].

Poverty, or a socioeconomically disadvantageous status, is a known risk factor for depression and anxiety symptoms among children [14–16]. Children whose mothers have been diagnosed with a major psychiatric disorder, also seem to present an increased risk for the development of mental problems [17], particularly for mother’s major depression disorder [18, 19]. Childhood obesity is another factor that might be associated with children mental health. Obesity may lead to depression in adolescents [20] and being overweighed or obese may be the leading reason for teasing and bullying among youth [21].

Mental health problems have also been associated with younger age groups (compared with adolescents) [22]. Mixed findings have been documented regarding sex, with evidence of more frequent mental problems occurring among boys [22] but also among girls [23].

Physical activity has also been associated with a lower likelihood of anxiety and depression symptoms [24]. However, studies using younger samples (including children) are needed, since the beneficial effect of physical activity over mental distress is not clear in younger ages [25].

Despite this knowledge, there is still need for better data gathering procedures and for the design of effective preventive actions tackling children mental health problems [26]. There are few studies involving reports of young children and considering several individual-level factors. Most studies depend on parents or other informants to assess child’s emotional state and behavior, increasing the propensity for socially desirable responses.

This study aims to explore several individual-level factors associated with self-reported symptoms of depression, anxiety and stress in a large sample of Portuguese children.

## Methods

### Participants and procedures

This study is part of a larger project entitled “Inequalities in Childhood Obesity: the impact of the socioeconomic crisis in Portugal from 2009 to 2015”. During 2016 and 2017, a total of 8472 school-aged children (mean age: 7.17 years, standard deviation: 1.91, 50.8% male), were recruited from 118 schools, public and private, in the cities of Porto, Coimbra and Lisbon. Participation rates were 60% in Porto, 58% in Coimbra and 67% in Lisbon. The same schools that participated in two previous projects were included. These projects were conducted during 2002 to assess childhood obesity prevalence [27], and during 2009 to assess changes in the prevalence of childhood obesity and the obesogenic environment [28].

Children attending pre-primary (if available in the school) and primary school were eligible to participate. Information letters describing the objective of the study were disseminated to all parents and written informed consent was asked for their participation and of their child. To parents of children aged 7.5 years and older, a specific additional consent was asked to allow their participation in a school-based survey, corresponding to the self-administration of a standardized questionnaire. The sample analyzed in this study is comprised of those who completed these self-administered questionnaires.

### Parents

Parents answered a standardized questionnaire comprising different sections that included: parental demographic and socioeconomic information, children lifestyle and parental common mental health symptoms.

For this study, father’s educational level was used as a proxy measure of socioeconomic status (SES), and categorized in low (corresponding to parents that had 9 years of school completed or less), medium (corresponding to parents that had secondary school level or from 10 to 12 years of school completed) and high (parents that had a university degree).

Parents’ self-reported height and weight was used to compute their Body Mass Index (kg/m^2^), which was subsequently categorized in normal, overweight or obese, according to the World Health Organization (WHO, 2000) guidelines for adults over 20 years old.

The adult version of the 21 item Portuguese Depression, Anxiety and Stress Scale – DASS [29, 30], was answered by the mother or the father (7 items for each subscale). Respondents were asked to refer to the previous week and provide their answer in a 4-option Likert scale (0 - Did not apply to me at all; 1 - Applied to me to some degree, or some of the time; 2 - Applied to me to a considerable degree, or a good part of time; 3 - Applied to me very much, or most of the time) regarding each item, which are statements representing specific negative emotional symptoms. Items are summed for each subscale, thus scores can vary between 0 and 21, with higher scores indicative of more symptoms.

### Children

Children date of birth and sex were reported by their parents and age was computed in years at the time of data collection.

Parents also answered whether their child participated in any organized (programmed and regular) sport activity other than the physical activity practiced at school (categorized as “yes/no”).

Children’s height and weight were objectively measured by trained researchers at school, in the morning, (SECA 220 scale and SECA 200 stadiometer) and BMI (as kg/m^2^) was subsequently categorized according to the International Obesity Task Force (IOTF) [31] cut-off points in normal weight, children with overweight and children with obesity.

Children aged 7.5 years or older, whose parents consented, were asked to answer a set of self-administered tools. The first question of the KIDSCREEN-27 questionnaire [32] was used as a measure of self-assessed health status – “In general, how would you describe your health: Excellent, Very Good, Good, Bad, Very Bad” – dichotomized in “Excellent or Very Good” and “Good, Bad or Very Bad” (only 0.5% of children reported “Bad” or “Very Bad” health status, hence this dichotomization).

Children were also asked to answer the child version of the 21-item DASS (the DASS-C) [33]. Conceptually, this questionnaire follows the exact same principles of the adult version, with language modifications to the items so these can be comprehended by children. The Portuguese version of the DASS-C has been validated using a student sample [34]. The questionnaires were handed over to teachers who, in turn, distributed them to children with parental permission to respond. Prior to the delivery of the questionnaires they were informed about each question by the researchers. All children with parental consent answered the questionnaire individually in the classroom. The teachers could enlighten the children about the understanding of each question if the child requested. Only questionnaires filled out by children, individually, in the school were accepted.

### Ethical approval

The study protocol was approved by Direção Geral do Ensino (Portuguese Ministry of Education) and Comissão Nacional de Proteção de Dados (CNPD), the Portuguese Data Protection Authority. All procedures were in accordance with the ethical standards of the Portuguese Data Protection Authority (Authorization number 745/2017) and with the 1964 Helsinki declaration and its later amendments or comparable ethical standards.

Written informed consent was obtained from children’s parents.

### Data analysis

Psychometric analysis of the DASS and DASS-C were conducted and are presented as Supplementary material (Tables 1, 2 and 3).

Out of the 8472 children assessed, 3957 were 7.5 years of age or older and 1469 provided full answers to the DASS-C questionnaire. A further 447 cases were excluded from the current analysis due to missing values in SES, mother BMI, DASS, if the DASS was not answered by the mother (mothers answered the DASS in more than 87% of cases), sports activity beyond that practiced at school and self-assessed health status. A total of 1022 cases were analyzed.

Compared to excluded children (Supplementary material – Table 4), those included in analysis presented a slightly higher mean age (8.77 vs. 8.62, *p* < 0.001), were more frequently females (52.9% vs. 47.5%, *p* = 0.003), were more frequently from Coimbra (53.7% of all included), practiced sport activity beyond school more often (72.3% vs. 61.2%, p < 0.001) and their mothers presented lower mean scores in the DASS Anxiety subscale (1.16 vs. 1.42, *p* = 0.009). Children classified with high socioeconomic status, were also more frequent among those included in analysis compared to those excluded (*p* = 0.005), although the proportions did not differ more than 5% between groups (Low = 24.5% vs. 28.7%; Medium = 36.8% vs. 37.9%; High = 38.7% vs. 33.3%). Due to these differences, it is not possible to state that this group of children constitutes a representative sample of Portuguese school-aged children.

Counts (and proportions), means (and standard deviations, sd) and medians (interquartile range, IQR) were calculated to describe categorical and continuous variables.

Each subscale of the DASS-C was categorized according to the quartiles of their distribution. Due to granularity in the data, the tertiles of the DASS subscales distribution were used for categorizing mothers’ DASS scores. Mean age was compared across the quartiles of the DASS-C subscales using ANOVA and the proportions of all other characteristics (sex, city of residence, children and mother’s BMI categories, socioeconomic status, sport activity beyond school, self-assessed health status and mother’s DASS tertiles scores) were compared across the quartiles of the DASS-C subscales using Chi-squared tests.

Then, each DASS-C subscale was dichotomized in quartile 4 (Q4) and quartiles 3, 2 or 1 (Q3, Q2 or Q1). The dichotomized variables were used as dependent variables in logistic regression (predicting the probability of scoring in Q4 vs. Q3, Q2 or Q1). Models were fitted for each individual variable and then fully adjusted. Crude and adjusted odds ratios (OR and AOR) and respective 95% confidence intervals (95%CI) were computed as measures of the magnitude of associations.

## Results

The psychometric analysis conducted to the DASS and DASS-C is presented as Supplementary material. In summary, the three solution factor (eigenvalue > 1) was confirmed for the DASS and also for the DASS-C. Cronbach’s Alphas for Depression, Anxiety and Stress subscales of the DASS in this sample were 0.84, 0.81 and 0.88, respectively, and the corresponding values for the DASS-C were 0.75, 0.73 and 0.78, respectively.

The sample characteristics are presented in Table 1. The mean age of the 1022 children analyzed was 8.77 years old (ranging from 7.5 to 11.5 years) and 52.9% were girls. The prevalence of children with overweight was 18.9% and there were 5.2% of children with obesity. Considering mother’s BMI categories, 24.1% were overweighed (without obesity) and 8.9% were obese. A total of 38.7% of fathers had a university degree (high socioeconomic status), and 72.3% of children practiced some type of organized sport activity (in a club or sports association) other than the physical activity practiced at school.

**Table 1:** Sample characteristics (*n* = 1022)

	*n* (%), or Mean (sd)	Median (IQR)	Min-Max
Age (years)	8.77 (0.77)	9.00 (1.50)	7.5–11.5
Sex			
Boys	481 (47.1)		
Girls	541 (52.9)		
City of residence			
Coimbra	549 (53.7)		
Lisbon	352 (34.4)		
Porto	121 (11.8)		
Children BMI (IOTF cut-offs)			
Normal	776 (75.9)		
Overweight	193 (18.9)		
Obesity	53 (5.2)		
Mother BMI (WHO cut-offs)			
Normal	685 (67.0)		
Overweight	246 (24.1)		
Obesity	91 (8.9)		
Socioeconomic status^a^ (Father education)			
Low	250 (24.5)		
Medium	376 (36.8)		
High	396 (38.7)		
Sport Activity beyond school			
Yes	739 (72.3)		
No	283 (27.7)		
Self Assessed Health Status			
Excellent, Very good	804 (78.7)		
Good, Bad, Very Bad	218 (21.3)		
Children DASS Depressive symptoms score	2.35 (2.87)	1.00 (3.25)	0–20
Children DASS Anxiety symptoms score	2.01 (2.63)	1.00 (3.00)	0–18
Children DASS Stress symptoms score	2.84 (3.09)	2.00 (4.00)	0–16
Mother DASS Depressive symptoms score	1.45 (2.41)	0.00 (2.00)	0–19
Mother DASS Anxiety symptoms score	1.16 (2.11)	0.00 (1.00)	0–18
Mother DASS Stress symptoms score	3.87 (3.31)	3.00 (5.00)	0–21

^a^Low socioeconomic status - if the father had 9 years of completed schooling or less; Medium socioeconomic status: if the father had between 10 and 12 years of completed schooling; High socioeconomic status – if the father had a university degree; *DASS* Depression, Anxiety and Stress scale, *IOTF* International obesity task-force, *WHO* World Health Organization, *sd* Standard deviation, *IRQ* Interquartile range;

Regarding their self-assessed health status, 78.7% of children declared their health was Excellent or Very Good, while the remaining 21.3% classified their health as Good, Bad or Very Bad (0.5% reported “Bad” or “Very Bad”).

Children mean (sd) scores in the DASS subscales were 2.35 (2.87) for Depressive symptoms, 2.01 (2.63) for Anxiety and 2.84 (3.09) for Stress. For mothers, these mean scores were 1.45 (2.41), 1.16 (2.11) and 3.87 (3.31), respectively.

Tables 2, 3 and 4 present the distribution of the children DASS-C Depressive, Anxiety and Stress scores, categorized in quartiles (higher scores correspond to most marked symptoms) according to the sample characteristics.

**Table 2:** Depressive symptoms subscale quartiles distribution according to sample characteristics

	DASS-C Depressive symptoms subscale score				
	Q1 (<=0.00)	Q2 (1.00–1.00)	Q3 (2.00–4.00)	Q4 (5.00 +)	*p*
*n*	352	178	307	185	
Age (continuous) [mean (sd)]	8.80 (0.72)	8.85 (0.79)	8.74 (0.78)	8.72 (0.81)	0.255
Sex [n(%)]					
Boys	143 (29.7)	82 (17.0)	153 (31.8)	103 (21.4)	
Girls	209 (38.6)	96 (17.7)	154 (28.5)	82 (15.2)	0.006
City of residence [n(%)]					
Coimbra	225 (41.0)	88 (16.0)	151 (27.5)	85 (15.5)	< 0.001
Lisbon	81 (23.0)	67 (19.0)	119 (33.8)	85 (24.1)	
Porto	46 (38.0)	23 (19.0)	37 (30.6)	15 (12.4)	
Children BMI (IOTF cut-offs) [n(%)]					
Normal	270 (34.8)	136 (17.5)	237 (30.5)	133 (17.1)	
Overweight	62 (32.1)	37 (19.2)	56 (29.0)	38 (19.7)	
Obesity	20 (37.7)	5 (9.4)	14 (26.4)	14 (26.4)	0.442
Mother BMI (WHO cut-offs)[n(%)]					
Normal	242 (35.3)	118 (17.2)	212 (30.9)	113 (16.5)	
Overweight	83 (33.7)	45 (18.3)	71 (28.9)	47 (19.1)	
Obesity	27 (29.7)	15 (16.5)	24 (26.4)	25 (27.5)	0.303
Socioeconomic status^a^ (Father education) [n(%)]					
Low	79 (31.6)	33 (13.2)	85 (34.0)	53 (21.2)	
Medium	133 (35.4)	66 (17.6)	106 (28.2)	71 (18.9)	
High	140 (35.4)	79 (19.9)	116 (29.3)	61 (15.4)	0.141
Sport Activity beyond school [n(%)]					
Yes	264 (35.7)	125 (16.9)	226 (30.6)	124 (16.8)	
No	88 (31.1)	53 (18.7)	81 (28.6)	61 (21.6)	0.212
Self Assessed Health Status [n(%)]					
Excellent, Very good	304 (37.8)	146 (18.2)	240 (29.9)	114 (14.2)	
Good, Bad, Very Bad	48 (22.0)	32 (14.7)	67 (30.7)	71 (32.6)	< 0.001
Mother DASS Depression score [n(%)]					
T1 (<=0.00)	200 (38.9)	90 (17.5)	147 (28.6)	77 (15.0)	
T2 (1.00–1.00)	67 (33.3)	33 (16.4)	62 (30.8)	39 (19.4)	
T3 (2.00 +)	85 (27.7)	55 (17.9)	98 (31.9)	69 (22.5)	0.027

*DASS-C* Depression, Anxiety and Stress scale – Children version; p – *p*-value from ANOVA (age) and Chi-square tests, *sd* Standard deviation, *BMI* Body Mass Index, *IOTF* International Obesity Taskforce, *WHO* World Health Organization, Q1, Q2, Q3, Q4 – Quartiles 1, 2, 3 and 4 of the DASS-C subscale score distribution; T1, T2, T3 – Tertiles 1, 2 and 3 of the DASS-C subscale score distribution

^a^Low socioeconomic status - if the father had 9 years of completed schooling or less; Medium socioeconomic status: if the father had between 10 and 12 years of completed schooling; High socioeconomic status – if the father had a university degree;

**Table 3:** Anxiety symptoms subscale quartiles distribution according to sample characteristics

	DASS-C Anxiety symptoms subscale score				
	Q1 (<=0.00)	Q2 (1.00–1.00)	Q3 (2.00–3.00)	Q4 (4.00 +)	*p*
*n*	381	202	228	211	
Age (continuous) [mean (sd)]	8.81 (0.73)	8.83 (0.79)	8.78 (0.81)	8.65 (0.80)	0.053
Sex [n(%)]					
Boys	160 (33.3)	93 (19.3)	117 (24.3)	111 (23.1)	
Girls	221 (40.9)	109 (20.1)	111 (20.5)	100 (18.5)	0.041
City of residence [n(%)]					
Coimbra	239 (43.5)	110 (20.0)	96 (17.5)	104 (18.9)	< 0.001
Lisbon	90 (25.6)	70 (19.9)	101 (28.7)	91 (25.9)	
Porto	52 (43.0)	22 (18.2)	31 (25.6)	16 (13.2)	
Children BMI (IOTF cut-offs) [n(%)]					
Normal	293 (37.8)	160 (20.6)	170 (21.9)	153 (19.7)	
Overweight	69 (35.8)	35 (18.1)	44 (22.8)	45 (23.3)	
Obesity	19 (35.8)	7 (13.2)	14 (26.4)	13 (24.5)	0.710
Mother BMI (WHO cut-offs) [n(%)]					
Normal	274 (40.0)	134 (19.6)	152 (22.2)	125 (18.2)	
Overweight	80 (32.5)	49 (19.9)	57 (23.2)	60 (24.4)	
Obesity	27 (29.7)	19 (20.9)	19 (20.9)	26 (28.6)	0.093
Socioeconomic status^a^ (Father education) [n(%)]					
Low	83 (33.2)	43 (17.2)	66 (26.4)	58 (23.2)	
Medium	147 (39.1)	73 (19.4)	72 (19.1)	84 (22.3)	
High	151 (38.1)	86 (21.7)	90 (22.7)	69 (17.4)	0.123
Sport Activity beyond school [n(%)]					
Yes	287 (38.8)	148 (20.0)	165 (22.3)	139 (18.8)	
No	94 (33.2)	54 (19.1)	63 (22.3)	72 (25.4)	0.103
Self Assessed Health Status [n(%)]					
Excellent, Very good	322 (40.0)	167 (20.8)	172 (21.4)	143 (17.8)	
Good, Bad, Very Bad	59 (27.1)	35 (16.1)	56 (25.7)	68 (31.2)	< 0.001
Mother DASS Anxiety score [n(%)]					
T1 (<=0.00)	254 (44.4)	108 (18.9)	107 (18.7)	103 (18.0)	
T2 (1.00–1.00)	65 (31.6)	50 (24.3)	55 (26.7)	36 (17.5)	
T3 (2.00 +)	62 (25.4)	44 (18.0)	66 (27.0)	72 (29.5)	< 0.001

*DASS-C* Depression, Anxiety and Stress scale – Children version, *p* p-value from ANOVA (age) and Chi-square tests, *sd* Standard deviation, *BMI* Body Mass Index, *IOTF* International Obesity Taskforce, *WHO* World Health Organization; Q1, Q2, Q3, Q4 – Quartiles 1, 2, 3 and 4 of the DASS-C subscale score distribution; T1, T2, T3 – Tertiles 1, 2 and 3 of the DASS-C subscale score distribution

^a^Low socioeconomic status - if the father had 9 years of completed schooling or less; Medium socioeconomic status: if the father had between 10 and 12 years of completed schooling; High socioeconomic status – if the father had a university degree;

**Table 4:** Stress symptoms subscale quartiles distribution according to sample characteristics

	DASS-C Stress symptoms subscale score				
	Q1 (<=0.00)	Q2 (1.00–2.00)	Q3 (3.00–5.00)	Q4 (6.00 +)	*p*
*n*	300	278	257	187	
Age (continuous) [mean (sd)]	8.86 (0.72)	8.78 (0.75)	8.71 (0.86)	8.70 (0.77)	0.074
Sex [n(%)]					
Boys	118 (24.5)	113 (23.5)	140 (29.1)	110 (22.9)	
Girls	182 (33.6)	165 (30.5)	117 (21.6)	77 (14.2)	< 0.001
City of residence [n(%)]					
Coimbra	180 (32.8)	165 (30.1)	124 (22.6)	80 (14.6)	< 0.001
Lisbon	77 (21.9)	79 (22.4)	104 (29.5)	92 (26.1)	
Porto	43 (35.5)	34 (28.1)	29 (24.0)	15 (12.4)	
Children BMI (IOTF cut-offs) [n(%)]					
Normal	224 (28.9)	230 (29.6)	186 (24.0)	136 (17.5)	
Overweight	62 (32.1)	37 (19.2)	53 (27.5)	41 (21.2)	
Obesity	14 (26.4)	11 (20.8)	18 (34.0)	10 (18.9)	0.075
Mother BMI (WHO cut-offs) [n(%)]					
Normal	212 (30.9)	180 (26.3)	171 (25.0)	122 (17.8)	
Overweight	70 (28.5)	76 (30.9)	57 (23.2)	43 (17.5)	
Obesity	18 (19.8)	22 (24.2)	29 (31.9)	22 (24.2)	0.164
Socioeconomic status^a^ (Father education) [n(%)]					
Low	70 (28.0)	61 (24.4)	63 (25.2)	56 (22.4)	
Medium	114 (30.3)	106 (28.2)	85 (22.6)	71 (18.9)	
High	116 (29.3)	111 (28.0)	109 (27.5)	60 (15.2)	0.269
Sport Activity beyond school [n(%)]					
Yes	228 (30.9)	203 (27.5)	183 (24.8)	125 (16.9)	
No	72 (25.4)	75 (26.5)	74 (26.1)	62 (21.9)	0.168
Self Assessed Health Status [n(%)]					
Excellent, Very good	258 (32.1)	230 (28.6)	191 (23.8)	125 (15.5)	
Good, Bad, Very Bad	42 (19.3)	48 (22.0)	66 (30.3)	62 (28.4)	< 0.001
Mother DASS Stress score [n(%)]					
T1 (<=2.00)	161 (38.8)	117 (28.2)	84 (20.2)	53 (12.8)	
T2 (3.00–5.00)	78 (25.1)	86 (27.7)	95 (30.5)	52 (16.7)	
T3 (6.00 +)	61 (20.6)	75 (25.3)	78 (26.4)	82 (27.7)	< 0.001

*DASS-C* Depression, Anxiety and Stress scale – Children version; *p* p-value from ANOVA (age) and Chi-square tests, *sd* Standard deviation, *BMI* Body Mass Index, *IOTF* International Obesity Taskforce, *WHO* World Health Organization; Q1, Q2, Q3, Q4 – Quartiles 1, 2, 3 and 4 of the DASS-C subscale score distribution; T1, T2, T3 – Tertiles 1, 2 and 3 of the DASS-C subscale score distribution

^a^Low socioeconomic status - if the father had 9 years of completed schooling or less; Medium socioeconomic status: if the father had between 10 and 12 years of completed schooling; High socioeconomic status – if the father had a university degree;

For the three DASS-C subscales quartiles, a statistically significant difference in the distribution of the sample proportions was found according to sex, city of residence, distribution of children self-assessed health status and distribution of the tertiles of mother’s DASS subscales score. There were slightly more girls than boys in the 1st quartile of the three DASS-C subscales and the opposite in the 4th quartile. Children from Lisbon (compared to Coimbra and Porto) scored more frequently in the 3rd and 4th quartiles of the three DASS-C subscales. A higher proportion of children reporting their health as Good, Bad or Very Bad was observed in the 4th quartile of the three DASS-C subscales. The proportion of the 3rd tertile of mother’s DASS answers was higher in the 4th quartile of children DASS-C subscales for the three subscales.

Table 5 shows the results from the logistic regression models fitted. After adjustment, age was negatively associated with increased DASS-C Anxiety symptoms score (AOR, 95%CI – Adjusted Odd Ratio, 95% Confidence Interval: 0.70, 0.57–0.87 for scoring in the 4th quartile vs. 3rd quartile or less). Compared with boys, girls presented lower odds of scoring in the 4th quartile of the DASS-C Depressive and Stress symptoms subscales, in the adjusted models (AOR, 95%CI: 0.65, 0.47–0.82 and 0.57, 0.41–0.80, respectively). Compared with Coimbra, children from Lisbon (but not from Porto) were more likely to report depressive, anxiety and stress symptoms (AOR, 95%CI: 1.75, 1.23–2.50; 1.55, 1.11–2.18; 2.16, 1.52–3.09, respectively).

**Table 5:** Factors associated with the DASS-C Depression, Anxiety and Stress subscales scores (dichotomized in Quartile 4 vs. Quartiles 3, 2 or 1 of the score distribution of each subscale)

		DASS-C Depressive Q4 (*n* = 185) vs. <Q3 (*n* = 837)		DASS-C Anxiety Q4 (*n* = 211) vs. <Q3 (*n* = 811)		DASS-C Stress Q4 (*n* = 187) vs. <Q3 (*n* = 835)	
*n*		OR (95%CI)	AOR (95%CI)	OR (95%CI)	AOR (95%CI)	OR (95%CI)	AOR (95%CI)
Age (continuous)	1022	0.89 (0.72–1.10)	0.83 (0.67–1.03)	0.76 (0.62–0.93)	0.70 (0.57–0.87)	0.86 (0.70–1.06)	0.82 (0.66–1.02)
Sex							
Boys	481	1	1	1	1	1	1
Girls	541	0.66 (0.48–0.90)	0.65 (0.47–0.92)	0.76 (0.56–1.02)	0.80 (0.58–1.10)	0.56 (0.41–0.77)	0.57 (0.41–0.80)
City of residence							
Coimbra	549	1	1	1	1	1	1
Lisbon	352	1.74 (1.24–2.43)	1.75 (1.23–2.50)	1.49 (1.08–2.06)	1.55 (1.11–2.18)	2.07 (1.48–2.90)	2.16 (1.52–3.09)
Porto	121	0.77 (0.43–1.39)	0.81 (0.44–1.47)	0.65 (0.37–1.15)	0.64 (0.36–1.15)	0.83 (0.46–1.50)	0.89 (0.48–1.63)
Children BMI (IOTF cut-offs)							
Normal	776	1	1	1	1	1	1
Overweight	193	1.19 (0.79–1.77)	0.94 (0.61–1.45)	1.24 (0.85–1.81)	1.04 (0.69–1.55)	1.27 (0.86–1.88)	1.09 (0.71–1.66)
Obesity	53	1.74 (0.92–3.29)	1.15 (0.57–2.32)	1.32 (0.69–2.54)	0.87 (0.43–1.77)	1.09 (0.54–2.23)	0.80 (0.36–1.75)
Mother BMI (WHO cut-offs)							
Normal	685	1	1	1	1	1	1
Overweight	246	1.20 (0.82–1.74)	1.12 (0.75–1.69)	1.45 (1.02–2.05)	1.39 (0.96–2.03)	0.98 (0.67–1.43)	0.93 (0.61–1.41)
Obesity	91	1.92 (1.16–3.17)	1.61 (0.93–2.80)	1.79 (1.09–2.94)	1.63 (0.96–2.77)	1.47 (0.88–2.47)	1.21 (0.68–2.13)
Socioeconomic status							
Low	250	1.48 (0.98–2.22)	1.22 (0.76–1.94)	1.43 (0.97–2.12)	1.10 (0.70–1.72)	1.62 (1.08–2.42)	1.61 (1.01–2.56)
Medium	376	1.28 (0.88–1.86)	1.24 (0.83–1.85)	1.36 (0.96–1.95)	1.32 (0.91–1.93)	1.30 (0.89–1.90)	1.33 (0.89–1.99)
High	396	1	1	1	1	1	1
Sport Activity beyond school							
Yes	739	1	1	1	1	1	1
No	283	1.36 (0.97–1.92)	1.13 (0.77–1.67)	1.47 (1.06–2.04)	1.34 (0.93–1.93)	1.38 (0.98–1.94)	1.26 (0.86–1.86)
Self-Assessed Health							
Excellent, Very good	804	1	1	1	1	1	1
Good, Bad, Very Bad	218	2.92 (2.07–4.13)	2.70 (1.87–3.91)	2.10 (1.49–2.94)	1.84 (1.28–2.65)	2.16 (1.52–3.07)	1.96 (1.34–2.88)
Mother DASS Depression score							
T1	514	1	1				
T2	201	1.37 (0.89–2.09)	1.38 (0.89–2.14)				
T3	307	1.65 (1.15–2.36)	1.67 (1.14–2.44)				
Mother DASS Anxiety score							
T1	572			1	1		
T2	206			0.96 (0.64–1.47)	0.91 (0.59–1.40)		
T3	244			1.91 (1.35–2.70)	1.74 (1.21–2.51)		
Mother DASS Stress score							
T1	415					1	1
T2	311					1.37 (0.91–2.08)	1.37 (0.89–2.09)
T3	296					2.62 (1.78–3.85)	2.75 (1.85–4.10)

*DASS-C* Depression, Anxiety and Stress scale – Children version, *BMI* Body Mass Index; *IOTF* International Obesity Taskforce, *WHO* World Health Organization; T1, T2, T3 – Tertiles 1, 2 and 3 of the DASS subscale score distribution

A low socioeconomic status, compared with a high socioeconomic status, was significantly associated with increased odds of scoring in the 4th quartile for the DASS-C Stress subscale (AOR, 95%CI: 1.61, 1.01–2.56).

Children who self-assessed their health as Good, Bad or Very Bad, compared with those assessing their health as Excellent or Very Good, showed significantly higher odds of scoring in the 4th quartile of the three DASS-C symptoms subscales, after adjustment (AOR, 95%CI for the DASS-C Depressive, Anxiety and Stress: 2.70, 1.87–3.91; 1.84, 1.28–2.65; 1.96, 1.34–2.88).

Increased odds of scoring in the 4th quartile (vs. 3rd or less) of the DASS-C Depressive, Anxiety and Stress subscales were found for the 3rd tertiles of the distribution of the mother’s scores (vs. 1st tertile) in the DASS Depressive, Anxiety and Stress subscales.

## Discussion

This exploration of the factors associated with mental health symptoms of young children suggests that girls, compared to boys, were less likely to report depression and stress symptoms and that children assessing their health as only Good, Bad or Very Bad, compared with those assessing their health as Excellent or Very Good, showed more frequent symptoms of depression, anxiety and stress. Children’ symptoms were also more frequent with the increase in mothers’ own reports on depressive, anxiety and stress symptoms.

### Interpretation of findings

#### Sex and age

The current findings suggest that boys tend to report higher levels of anxiety and depressive symptoms than girls. This has also been suggested in a recent Iranian study where more common mental health problems were found among boys [22]. However, in a large community sample of primary school aged children (8–12 years) from Singapore (*n* = 1655), girls reported more anxiety and depressive symptoms than boys [23]. Sex differences in the expression of these symptoms might be influenced by the cultural context, and might be less noticeable in younger age groups, thus becoming more pronounced with increasing age.

In a meta-analytic study of gender differences in emotion expression of children, it was found that girls show more positive emotions than boys during childhood, while boys show more externalizing emotions [35]. These differences were context-dependent for internalizing, externalizing and negative emotion expressions, and may depend on different tendencies of boys and girls to provide answers that conform to societal gender roles [35]. In our sample, such tendency might have influenced girls’ responses and explain the observed sex-differences.

#### Place of residence

Children from Lisbon reported symptoms of common mental health more frequently than children from Coimbra or Porto. There may be specific features of Lisbon’s urban environment which increase children likelihood of depressive, anxiety and stress symptoms. Lisbon metropolitan area is the country’s most densely populated region, where people travel longer daily distances. One hypothesis that could explain these results is that in Lisbon children spend more time in traffic to and from school, which can affect negatively both mother and child anxiety, depressive and stress symptoms. Further comparisons within and across cities are needed to disentangle the individual effect of environmental characteristics on mental health symptoms of children, including the time commuting to school. Future studies should also consider the effect of living in a disadvantaged or deprived neighborhood, since it has been associated with poor mental health outcomes [36].

#### Body mass index

A significant association between children and mother’s BMI categories with depression, anxiety and stress symptoms was not found in this study. However, the point estimates obtained, particularly for mother’s BMI, suggest a graded influence on child symptoms. Given the association of mother’s BMI on children BMI [37, 38], the physical health burden associated with overweight and obesity [39], and the potential bullying and stigma experienced by overweight/obese children [21], interventions aiming to promote mental health among children should include strategies that also aim to prevent excessive weight gain.

#### Socioeconomic status

The consistent association found between parental socioeconomic status and children mental health, suggests that children of less educated fathers reported more frequent symptoms of common mental problems. This is in line with the results from other studies [14–16]. Growing up in a disadvantaged environment has been associated with several poor health outcomes [40], thus, this particular result highlights the need to address socioeconomic disparities in Portuguese children from an early stage.

#### Sport activity

We did not find a significant association between mental health symptoms and sport activity outside school, although physical activity has been identified as a protective factor of poor mental health [41, 42]. Physical activity may act through physiological changes (decreases in cortisol levels) [43], psychological changes (e.g. reducing anger, fatigue) [44] and social changes (e.g. enabling opportunities for social support), thus reducing or preventing anxiety and depression symptoms. More detailed information about sports activity engagement among Portuguese primary-school aged children is needed to measure its effect on mental health. Nevertheless, given the documented benefits for health and the fact that Portuguese children in this age range present low levels of physical activity [45], sport activity should be promoted in early school years.

#### Mother’s mental health symptoms

The influence of mother’s depressive symptoms on children’s negative emotions has been a topic of great concern [46, 47], but less is known regarding anxiety and stress, although anxiety is considered a very common condition in pre-adolescent ages [6].

A recent study in Uganda showed that caregiver anxiety, depressive symptoms and low social support, were associated with worse well-being in school-aged and adolescent children [48]. Depressive symptoms in mothers might limit their functioning, increase children’s behavioral and developmental problems [49, 50].

Mother’s negative emotions may influence child emotional and behavioral adjustment at very early stages, and a bidirectional influence (with the child’s own symptoms influencing mother’s emotion) might explain the congruence between mother-child depressive symptoms [51]. Other unmeasured factors may also play a role in the relation between mother-child mental health symptoms, namely poor parent-child attachment or inadequate supervision. These factors would be better explored using a longitudinal design [52].

#### Strengths and limitations

The appropriateness of assessing depression, anxiety and stress symptoms among children, specifically using the DASS-C, has been questioned, since these symptoms might be indistinguishable in children and adolescents [53]. In the scales’ original psychometric assessment [33], only the stress subscale was pointed out as needing refinement. The Portuguese validation of the children version of the scale was conducted with a smaller (*n* = 361) sample of children slightly older compared to the children enrolled in our study (children aged 8 to 15 years old). In the validation study, the scale was individually filled, although administration occurred in collective and individual sessions [34], and lower correlations were also found for the stress subscale [34]. In this validation study, no differences in the results were found when the sample was stratified by age in younger (8–11 years) and older (12–15 years) children [34]. In the present study the factorial analysis of the DASS and the DASS-C suggests confirmation of the 3-factor solution for both versions (Supplementary material). Nevertheless, future studies should test the scale’s reliability since few studies have documented this scale’s performance in younger samples. Poor psychometric characteristics have also been found, particularly for the Anxiety and Stress subscales of the DASS-C [53]. For this reason, the results presented for these subscales should be interpreted with caution.

Assessing this type of variables in young children might be particularly difficult considering their literacy limitations, although possible with children aged 7 years and older and with a sixth-grade reading level or higher [54], which might have not been the case with the current sample. For this reason, the current results must also be interpreted with caution.

The current study did not explore the role of parental psychiatric condition as an associated factor with children’s mental health symptoms, nor the history of any traumatic incident or other adverse childhood events, including child maltreatment [55]. Since the tertiles of the distribution of mothers’ DASS scores were used as an exposure measure, and given the skewed distribution of such scores, these results show the probability of children declaring more symptomatology even for very low scores of mother’s symptoms.

A major strength of the current study lies in children’s self-reported outcome measure: children answered the DASS-C at school without parental influence. Previous research assessing mental health of primary school-aged children documents parental reports to specific questionnaires and not actual child answers, which are harder to obtain. In addition, parental reports of children mental health status may be biased, particularly, if mothers are experiencing depression [49].

Finally, due to the differences observed between included and excluded children (more girls, slightly older, more frequently from Coimbra and more frequently classified with high socioeconomic status were included), it is not possible to state that this group of children constitutes a representative sample of Portuguese school-aged children and, for this reason, the results should not be generalized.

## Conclusion

The potential negative developmental consequences associated with childhood depressive, anxiety and stress symptoms should be prevented. This study suggests that place of residence, individual characteristics and parental factors, in specific the depressive, anxiety and stress symptoms of the mother, are important modifiable factors that must be considered in the development of contextualized preventive efforts for childhood mental problems.

## Supplementary information

**Additional file 1: Table S1**. Principal component analysis: total variance explained for DASS and DASS-C. **Table S2**. Rotated Component Matrix (Varimax) for the DASS and DASS-C from Principal Component Analysis. **Table S3**. Cronbach alphas for DASS and DASS-C. **Table S4**. Characteristics of Included vs. Excluded children in analysis.

## Data Availability

The datasets used and/or analysed during the current study are available from the corresponding author on reasonable request.

## Abbreviations

AOR: Adjusted Odds Ratio
BMI: Body Mass Index
CI: Confidence Interval
CNPD: Comissão Nacional de Proteção de Dados
DASS: Depression, Anxiety and Stress Scale
DASS-C: Depression, Anxiety and Stress Scale - Children
IOTF: International Obesity Task force
IQR: Interquartile Range
OR: Odds Ratio
Q1, Q2, Q3, Q4: Quartiles 1, 2, 3, 4
SES: Socioeconomic Status
