# Supplementary material for: Self-reported symptoms of depression, anxiety and stress in Portuguese primary school-aged children
Source: BMC Psychiatry. 2020 Feb 27;20:87. doi: 10.1186/s12888-020-02498-z (PMC7047394; doi:10.1186/s12888-020-02498-z)
Supplement: Supplementary file 1 — Additional file 1: Table S1. Principal component analysis: total variance explained for DASS and DASS-C. Table S2. Rotated Component Matrix (Varimax) for the DASS and DASS-C from Principal Component Analysis. Table S3. Cronbach alphas for DASS and DASS-C. Table S4. Characteristics of Included vs. Excluded children in analysis. [file 12888_2020_2498_MOESM1_ESM.docx]

Table S1. Principal component analysis: total variance explained for DASS and DASS-C

|  | **DASS** | | |  | **DASS-C** |  |
| --- | --- | --- | --- | --- | --- | --- |
| **Component** | **Initial Eigenvalues** | | | **Initial Eigenvalues** | | |
|  | **Total** | **% of Variance** | **Cumulative %** | **Total** | **% of Variance** | **Cumulative %** |
| 1 | 8.620 | 41.048 | 41.048 | 6.794 | 32.351 | 32.351 |
| 2 | 1.658 | 7.897 | 48.945 | 1.067 | 5.080 | 37.431 |
| 3 | 1.198 | 5.704 | 54.648 | 1.037 | 4.940 | 42.371 |
| 4 | 0.878 | 4.181 | 58.829 | 0.994 | 4.732 | 47.103 |
| 5 | 0.784 | 3.733 | 62.562 | 0.894 | 4.258 | 51.361 |
| 6 | 0.758 | 3.610 | 66.172 | 0.867 | 4.131 | 55.492 |
| 7 | 0.673 | 3.206 | 69.378 | 0.817 | 3.890 | 59.382 |
| 8 | 0.637 | 3.033 | 72.411 | 0.790 | 3.761 | 63.143 |
| 9 | 0.583 | 2.774 | 75.185 | 0.775 | 3.690 | 66.833 |
| 10 | 0.567 | 2.702 | 77.887 | 0.736 | 3.505 | 70.338 |
| 11 | 0.556 | 2.647 | 80.533 | 0.683 | 3.253 | 73.591 |
| 12 | 0.515 | 2.454 | 82.987 | 0.660 | 3.143 | 76.734 |
| 13 | 0.486 | 2.316 | 85.304 | 0.633 | 3.014 | 79.748 |
| 14 | 0.444 | 2.115 | 87.418 | 0.598 | 2.847 | 82.595 |
| 15 | 0.419 | 1.997 | 89.415 | 0.584 | 2.780 | 85.375 |
| 16 | 0.407 | 1.940 | 91.355 | 0.558 | 2.656 | 88.031 |
| 17 | 0.398 | 1.893 | 93.249 | 0.553 | 2.635 | 90.666 |
| 18 | 0.374 | 1.779 | 95.028 | 0.525 | 2.498 | 93.164 |
| 19 | 0.363 | 1.726 | 96.754 | 0.511 | 2.433 | 95.597 |
| 20 | 0.361 | 1.719 | 98.473 | 0.485 | 2.312 | 97.909 |
| 21 | 0.321 | 1.527 | 100.000 | 0.439 | 2.091 | 100.000 |

Table S2. Rotated Component Matrix (Varimax) for the DASS and DASS-C from Principal Component Analysis

|  | **DASS** | | | **DASS-C** | | |
| --- | --- | --- | --- | --- | --- | --- |
|  | **Stress** | **Depression** | **Anxiety** | **Stress** | **Depression** | **Anxiety** |
| Item 1 | 0.696 | 0.129 | 0.220 | 0.673 | 0.154 | 0.200 |
| Item 2 | 0.303 | 0.160 | 0.452 | 0.239 | 0.043 | 0.527 |
| Item 3 | 0.173 | 0.526 | 0.157 | 0.214 | 0.331 | 0.302 |
| Item 4 | 0.107 | 0.083 | 0.713 | 0.240 | 0.153 | 0.471 |
| Item 5 | 0.397 | 0.446 | 0.087 | 0.149 | 0.135 | 0.581 |
| Item 6 | 0.722 | 0.119 | 0.093 | 0.644 | 0.084 | 0.150 |
| Item 7 | 0.171 | 0.202 | 0.666 | 0.133 | 0.337 | 0.516 |
| Item 8 | 0.723 | 0.139 | 0.310 | 0.460 | 0.377 | 0.376 |
| Item 9 | 0.255 | 0.283 | 0.560 | 0.382 | 0.483 | 0.010 |
| Item 10 | 0.192 | 0.743 | 0.212 | 0.151 | 0.497 | 0.289 |
| Item 11 | 0.698 | 0.168 | 0.346 | 0.560 | 0.237 | 0.254 |
| Item 12 | 0.724 | 0.226 | 0.251 | 0.522 | 0.274 | 0.339 |
| Item 13 | 0.538 | 0.519 | 0.204 | 0.156 | 0.626 | 0.294 |
| Item 14 | 0.602 | 0.321 | 0.149 | 0.556 | 0.341 | 0.091 |
| Item 15 | 0.246 | 0.403 | 0.584 | 0.310 | 0.613 | 0.130 |
| Item 16 | 0.246 | 0.730 | 0.248 | 0.046 | 0.173 | 0.662 |
| Item 17 | 0.224 | 0.746 | 0.226 | 0.234 | 0.569 | 0.224 |
| Item 18 | 0.660 | 0.296 | 0.193 | 0.133 | 0.637 | 0.192 |
| Item 19 | 0.289 | 0.225 | 0.642 | 0.175 | 0.329 | 0.548 |
| Item 20 | 0.204 | 0.435 | 0.577 | 0.121 | 0.682 | 0.106 |
| Item 21 | 0.087 | 0.767 | 0.297 | 0.147 | 0.517 | 0.384 |
|  | | | |  |  |  |

Table S3. Cronbach alphas for DASS and DASS-C.

| **Subscale** | **DASS** | **DASS-C** |
| --- | --- | --- |
| **Stress** | 0.875 | 0.777 |
| **Depression** | 0.844 | 0.751 |
| **Anxiety** | 0.814 | 0.728 |
| **Total** | 0.923 | 0.893 |

Table S4. Characteristics of Included vs. Excluded children in analysis

|  |  | | **Included (n=1022)** | **Excluded (n=2935)** |  |
| --- | --- | --- | --- | --- | --- |
|  |  | | **n (%),**  **or Mean (sd)** | **n (%),**  **or Mean (sd)** | **p-value** |
| **Age (years)** |  | | 8.77 (0.77) | 8.62 (0.87) | <0.001 |
| **Sex** | **Boys** | | 481 (47.1) | 1541 (52.5) |  |
|  | **Girls** | | 541 (52.9) | 1394 (47.5) | 0.003 |
| **City of residence** | **Coimbra** | | 549 (53.7) | 925 (31.5) |  |
|  | **Lisbon** | | 352 (34.4) | 1232 (42.0) |  |
|  | **Porto** | | 121 (11.8) | 778 (26.5) | <0.001 |
| **Children BMI**  **(IOTF cut-offs)** | **Normal** | | 776 (75.9) | 2160 (73.6) |  |
|  | **Overweight** | | 193 (18.9) | 575 (19.6) |  |
|  | **Obesity** | | 53 (5.2) | 200 (6.8) | 0.143 |
| **Mother BMI**  **(WHO cut-offs)** | **Normal** | | 685 (67.0) | 1409 (64.3) |  |
|  | **Overweight** | | 246 (24.1) | 549 (25.1) |  |
|  | **Obesity** | | 91 (8.9) | 233 (10.6) | 0.209 |
| **Socioeconomic status***  **(Father education)** | **Low** | | 250 (24.5) | 653 (28.7) |  |
|  | **Medium** | | 376 (36.8) | 862 (37.9) |  |
|  | **High** | | 396 (38.7) | 758 (33.3) | 0.005 |
| **Sport Activity beyond school** | **Yes** | | 739 (72.3) | 1517 (61.2) |  |
|  | **No** | | 283 (27.7) | 963 (38.8) | <0.001 |
| **Self-Assessed Health Status** | **Excellent, Very good** | | 804 (78.7) | 434 (76.8) |  |
|  | **Good, Bad, Very Bad** | | 218 (21.3) | 131 (23.2) | 0.429 |
| **Mother DASS Depressive symptoms score** | |  | 1.45 (2.41) | 1.56 (2.66) | 0.310 |
| **Mother DASS Anxiety symptoms score** | |  | 1.16 (2.11) | 1.42 (2.48) | 0.009 |
| **Mother DASS Stress symptoms score** | |  | 3.87 (3.31) | 3.72 (3.51) | 0.309 |

*Low socioeconomic status - if the father had 9 years of completed schooling or less; Medium socioeconomic status: if the father had between 10 and 12 years of completed schooling; High socioeconomic status – if the father had a university degree; DASS – Depression, Anxiety and Stress scale; IOTF – International obesity task-force; WHO – World Health Organization; sd – standard deviation; p-value from Chi-squared test comparing proportions or T-test comparing means.
